# Patient Perception of Providers: Do Patients Understand Who Their Doctor Is?

**DOI:** 10.1177/2374373519892780

**Published:** 2019-12-04

**Authors:** Alisa Wray, Maja Feldman, Shannon Toohey, Andrew Wong, Wynne Breed, Emily Frisch, Soheil Saadat, Warren Wiechmann

**Affiliations:** 1 Department of Emergency Medicine, 8788 University of California, Irvine, CA, USA; 2 Department of Emergency Medicine, 8788 University of California, Davis, CA, USA

**Keywords:** medical provider role, patient perspectives, narratives, emergency medicine, clinician-patient relations

## Abstract

**Background:**

When being treated at a university-based hospital, a patient may encounter multiple levels of physicians, including trainees during a single emergency visit. Patients want to know the roles of their providers, but their understanding of the medical education hierarchy is poor.

**Objectives:**

Our study explored patient understanding of commonly used physician and trainee titles as well as the factors that contribute to patient understanding in our emergency department patient population. Additionally, we evaluated a new badge buddy system that identifies medical personnel impacts patient’s perceptions of providers. We examined how the increasing prevalence of medicine in media may change patient perceptions of the medical hierarchy.

**Methods:**

Patients pending discharge from the emergency room was assessed through a knowledge-based and opinion-based questionnaire. Questions quantified the percentage of patients who understood titles of their team.

**Results:**

Of 423 patients who completed the study, 88% (N = 365) felt it was very important to know the level of training of their doctor when being treated in the emergency department. Seventy-four percent (N = 303) believed they knew the role of their care providers but the mean knowledge score was 4.7 of 8, suggesting a poor understanding of the medical training hierarchy. Younger patients and those who felt that knowing the level of training of their doctor was very important noticed the badge buddies more frequently (80.9%, *P* = .020 and 81%, *P* < .001).

**Conclusions:**

Our study found that patients had a poor understanding of the medical training hierarchy, but felt that it is important to know the level of training of their staff. The implementation of a badge buddy served this purpose for most patients, but was less effective for older patients. Further research may be needed to evaluate if a different intervention, such as a detailed video or teach-back techniques explaining the levels of medical training, would be more effective for a larger population of patients.

## Introduction

Patients presenting to the emergency department (ED) at a university-based teaching hospital are taken care of by medical staff at many levels of training. From the medical student to attending physician, a patient may encounter multiple levels of physicians including trainees during 1 emergency visit. Prior studies have demonstrated that patients believe that knowledge of a physician’s level of training as important [1–3]. However, studies show patient’s understanding of the medical education hierarchy is generally poor [4–6]. For example, one study found that respondents accurately identified the level of training of physicians and students in only 44.5% of surveys collected [6]. While in another, 33% of respondents did not know that attending physicians supervise the interns [2].

This lack of understanding could lead to several issues in the patient encounter. First, interactions with multiple providers could cause frustration and decreased satisfaction for patients who do not understand different levels of trainees evaluating them during their visit [6]. Furthermore, misunderstanding of roles could affect patient safety or the ability to consent to treatment if they are given information by a trainer believing it to be the attending physician. Previous studies have shown that increased patient knowledge is associated with increased comfort with being treated by a physician in training [4].

Our study explored patient understanding of commonly used physician and trainee titles as well as the factors that contribute to patient understanding in our ED patient population. We sought to evaluate patient understanding of these titles. Additionally, we evaluated whether a new badge buddy system (an additional badge connected to the name badge), which clearly identifies medical personnel as medical student, resident or attending physician, impacted our patients’ perceptions of their providers. Lastly, we explored whether the increasing prevalence of medical dramas and reality shows contributes to patient understanding of the educational hierarchy of medical professionals.

## Methodology

In 2016, the University of California, Irvine Medical Center, implemented a “badge buddy” system, with a large blue tag attached to name tags clearly identifying patient care providers with terms such as “medical student,” “resident,” and “attending” that have specific meaning and define the commonly understood hierarchy associated with medical education. After the implantation of this system, we collected survey responses from 423 adult patients in the ED regarding their knowledge of the medical education hierarchy. The data were collected via convenience sample over a 2-month period. Our ED has a census of 56 000 per year, with 94% of patients being adults, so had an estimate of approximately 8773 adult patients during this same period. So our response rate was approximately 5%. The survey questions aimed to quantify the percentage of our ED patients who could correctly answer knowledge-based questions on patient understanding of commonly used physician and student titles of their health-care team. Questions were created based on literature review of previous similar studies [1–6] and questions were then reviewed and edited by the physicians involved in the study for clarity and understanding. We collected demographic information, specifically age, gender, race/ethnicity, frequency of ED visits, and level of education. We also collected responses to yes/no questions and Likert scale questions which can be found in Supplement 1.

This study was approved by the University of California, Irvine Institutional Review Board. We performed a cross-sectional analysis of survey data collected from patients in our urban, 40-bed, University-based ED. The study included English-speaking patients elder than 18 years, who were in stable condition and pending discharge. Patients were approached by a member of an undergraduate survey team, Emergency Medicine Research Apprenticeship Program, with an electronic survey. Patients willing to participate were verbally consented and no personal identifying information was collected. As these data were collected prospectively, no a priori sample size was calculated. The survey was collected anonymously on an iPad via PollDaddy.com and took approximately 10 minutes to complete. The survey consisted of 8 multiple-choice knowledge-based questions, 10 opinion-based questions using a 4-point Likert scale, and 6 demographics questions.

The knowledge-based questions were scored by assigning 1 to correct and 0 to wrong and “I don’t know” choices. We calculated a “knowledge score” for every responder by adding up the scores, they attained in knowledge-based questions (theoretical range: 0-8). We recorded the opinion-based questions into dichotomous variables by combining “agree” and “strongly agree” choices into a single category and also combining “do not agree” and “strongly do not agree” choices into another single category. The distribution of categorical variables are presented as N (%) and the distribution of knowledge score is presented as mean, standard deviation (SD), median, and interquartile range (IQR). We used Chi-square statistics to compare the distribution of opinion-based questions against different groups, and the “independent samples Kruskal-Wallis” or “independent samples Mann-Whitney *U*” test to compare the distribution of knowledge score. A *P* value of less than .05 was considered statistically significant. IBM SPSS Statistics version 25 was used to perform the analysis.

## Results

### Patients Characteristics

**Figure 1.**
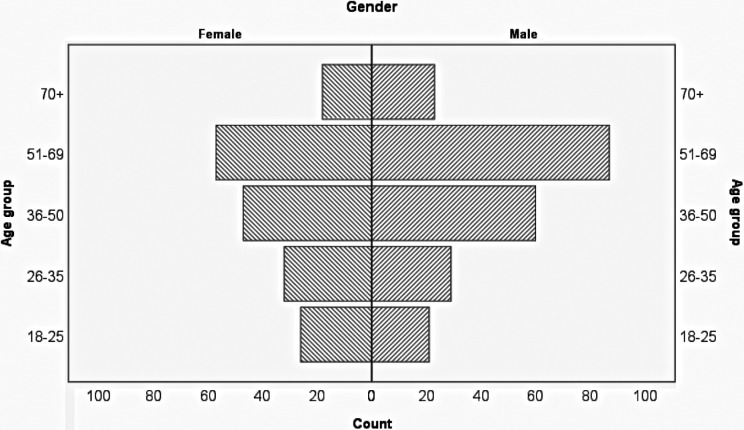
Age and gender distribution of study participants.

Figure 1 shows the age and gender distribution of study sample. Table 1 shows the demographics of all respondents.The education level of 148 (37.6%) patients was high school or less. White (N = 151, 43.1%) followed by Hispanic/Latino (N = 138, 39.4%) were the most common race/ethnicity among responders. The range of incomes was broad and while the annual income of 174 (54.5%) patients was less than $25 000, 37 (11.6%) participants had earned more than $100 000 during the past year. It was the first self-reported visit to the ED during the past 12 months for 160 (40.7%) patients, the second visit for 93 (23.7%) patients, while 42 (10.7%) had 3 or more visits in the past 12 months.

**Table 1.** Characteristics of Study Participants.^a^

Characteristics Levels		n	Proportion (%)
Age	18-25	47	11.8
	26-35	61	15.3
	36-50	107	26.8
	51-69	144	36.0
	70+	41	10.3
Gender	Female	181	45.0
	Male	221	55.0
Education level	High school or less	148	37.6
	Above high school	246	62.4
Race/ethnicity	White	151	43.1
	Hispanic/Latino	138	39.4
	Asian	40	11.4
	African American	21	6.0
Income levels	$0-$24 999	174	54.5
	$25 000-$49 000	57	17.9
	$50 000-$74 999	36	11.3
	$75 000-$99 000	15	4.7
	$100 000 +	37	11.6
N of ED visits in the past 12 months	1	160	40.7
	2	93	23.7
	3	42	10.7
	4-5	50	12.7
	> 6	48	12.2

Abbreviation: ED, emergency room.

^a^ Percentages may not add up to exactly 100 due to rounding up. n may be less than total N of 423 as some participants declined to answer some questions.

### Knowledge of the Medical Education Hierarchy

**Figure 2.**
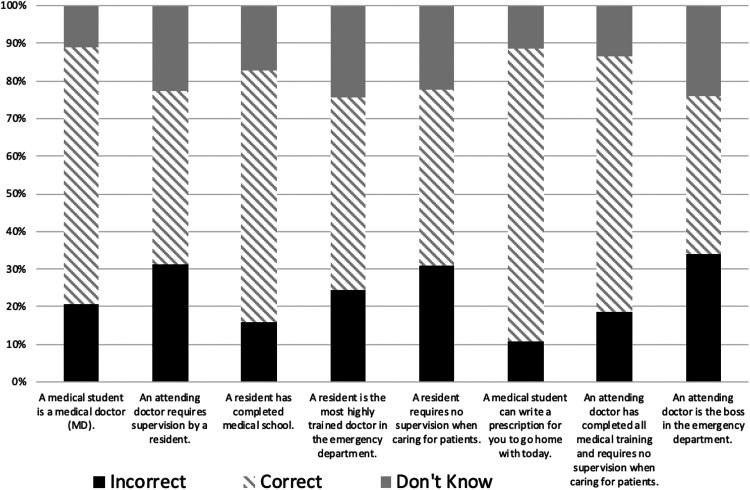
Distribution of incorrect answers regarding medical education hierarchy.

The mean knowledge score of the respondents was 4.7 of 8 (SD: ±1.96) with a median of 5 (IQR: 15 percentile: 3.0; 75 percentile 6.0). Figure 2 shows the distribution of incorrect answers among the knowledge-based questions.

The questions with the most frequent incorrect responses were “an attending doctor is the boss in the emergency department” (34.0%; N = 144) followed by “an attending doctor requires supervision by a resident” (31.4%; N = 133) and “a resident requires no supervision when caring for patients” (30.7%; N = 130).

The question with the highest percent of correct answers was “a medical student can write a prescription for you to go home with today” with 77.5% (N = 328) of respondents answering no.

Table 2 shows the association of knowledge score with patients’ characteristics. The knowledge score was associated with education of more than high school (*P* < .001) and higher income (*P* = .001) and it was highest among the white patients (*P* < .001).

**Table 2.** Comparison of Knowledge Score by Patients’ Characteristics.^a^

		n	Mean	Standard Deviation	Median	*P* Value
Age groups 35	18-35 years	47	4.30	2.05	4.00	.194
	>36 years	312	4.74	1.89	5.00	
Gender	Female	181	4.86	1.93	5.00	.146
	Male	221	4.59	1.88	5.00	
Education level	High school or less	148	3.97	1.79	4.00	<.001
	Above high school	246	5.22	1.79	5.00	
Income levels	$0-$24 999	174	4.47	1.91	5.00	.001
	$25 000-$49 000	57	4.61	1.87	4.00	
	$50 000-$74 999	36	5.08	1.79	5.00	
	$75 000-$99 000	15	5.20	1.52	5.00	
	$100 000 +	37	5.78	1.97	6.00	
Race/ethnicity	Asian	40	4.93	1.76	5.00	<.001
	Black/African American	21	4.86	1.46	5.00	
	Hispanic/Latino	138	4.01	1.82	4.00	
	White	151	5.39	1.85	5.00	
ED visits during past year	1	160	4.66	1.88	5.00	.254
	2	93	5.15	1.70	5.00	
	3	42	4.57	2.01	5.00	
	4-5	50	4.68	2.01	5.00	
	>6	48	4.48	2.02	4.00	

Abbreviation: ED, emergency room.

^a^ Kruskal-Wallis test used for race and income level, Mann-Whitney *U* test for education level.

### Patients’ Opinion About Medical Providers

In the opinion-based questions, 88.0% (N = 365) of respondents felt it was very important to know the level of training of their doctor when being treated in the ED. Seventy-four percent (N = 303) believed they knew the roles of their care providers. Sixty-eight percent (N = 273) felt that the badge buddy helped them figure out the role of their medical provider. Table 3 shows the complete opinion-based results.

**Table 3.** Opinion of Patients on Medical Training Hierarchy and Medical Providers Identification.^a^

Questions	Opinions			
	Strongly Agree n (%)	Agree n (%)	Do Not Agree n (%)	Strongly Do Not Agree n (%)
1. It is very important to me to know the level of training of my doctor when I am being treated in the ED	182 (43.9)	183 (44.1)	34 (8.2)	16 (3.9)
2. I knew the roles of my care providers	118 (28.7)	185 (45.0)	93 (22.6)	15 (3.6)
3. The medical provider who cared for me identified him/herself	141 (34.2)	173 (42.0)	75 (18.2)	23 (5.6)
4. I could clearly see this badge on my medical providers	173 (43.3)	144 (36.0)	70 (17.5)	13 (3.3)
5. My medical providers wore this badge	179 (45.1)	152 (38.3)	59 (14.9)	7 (1.8)
6. I was satisfied with the amount of information my providers gave to me regarding their level of training	137 (34.3)	176 (44.1)	78 (19.5)	8 (2.0)
7. This badge helped me figure out the role of my medical providers	138 (34.6)	135 (33.8)	115 (28.8)	11 (2.8)
8. In the past, I have frequently watched medical shows on TV	104 (25.9)	129 (32.1)	103 (25.6)	66 (16.4)
9. Overall, I was satisfied with my visit today and the care I received by my medical team	187 (46.8)	188 (47.0)	18 (4.5)	7 (1.8)

Abbreviation: ED, emergency room.

^a^ n may be less than total N of 423 as some participants declined to answer some questions.

### Factors Associated With “Seeing” the Badge Buddy

Patients variably noticed the badge buddy worn by care providers. Although 85.6% (N = 298) of the patients aged 18 to 69 noticed the badge buddy, only 70.0% (N = 28) of patients aged 70+ noticed it (*P* = .020). Moreover, patients who answered that knowing the level of training of their doctors was very important, more frequently noticed the badge buddy (*P* < .001) and also more frequently answered that they were able to clearly see the badge (*P* = .022) (Table 4). Watching medical shows was also associated with higher remembrance of badge (*P* = .006) and reporting to have seen the badge (*P* = .033; Table 4).

**Table 4.** Patients’ Characteristics Associated With Paying Attention to Badges Worn by Providers.^a^

Characteristics		I Could Clearly See the Badge					My Medical Providers Wore the Badge				
		Level					Level				
		Agree		Disagree		*P* Value	Agree		Disagree		*P* Value
		N	(%)	N	(%)		N	(%)	N	(%)	
Age Group (70)	18-69	284	(80.9)	67	(19.1)	.094	298	(85.6)	50	(14.4)	.020^b^
	70+	27	(69.2)	12	(30.8)		28	(70.0)	12	(30.0)	
Education level	High school or less	115	(79.9)	29	(20.1)	>.999	119	(82.6)	25	(17.4)	.379
	Above high school	192	(80.0)	48	(20.0)		206	(86.2)	33	(13.8)	
Income levels	$0-$24 999	133	(78.2)	37	(21.8)	.241	141	(83.9)	27	(16.1)	.157
	$25 000-$49 000	48	(87.3)	7	(12.7)		49	(89.1)	6	(10.9)	
	$50,000-$74,999	30	(85.7)	5	(14.3)		31	(88.6)	4	(11.4)	
	$75 000-$99 000	13	(86.7)	2	(13.3)		15	(100.0)	0	(0.0)	
	$100 000 +	31	(83.8)	6	(16.2)		32	(88.9)	4	(11.1)	
It is very important to me to know the level of training of my doctor	Agree	285	(81.0)	67	(19.0)	.029^c^	301	(86.2)	48	(13.8)	<.001^d^
	Disagree	31	(66.0)	16	(34.0)		29	(61.7)	18	(38.3)	
I have frequently watched medical shows	Agree	190	(83.0)	39	(7.0)	.040^e^	197	(87.9)	27	(12.1)	.007^f^
	Disagree	122	(73.9)	43	(26.1)		129	(77.2)	38	(22.8)	

^a^ n may be less than total N of 423 as some participants declined to answer some questions. Chi-square test used to compare variables.

^b^ Chi-square = 5.42.

^c^ Chi-square = 5.67.

^d^ Chi-square = 17.97.

^e^ Chi-square = 4.21.

^f^ Chi-square = 7.15.

### Factors Associated With Finding the Badges Useful and the Satisfaction With the Amount of Information Received

Badges were assessed more useful by patients aged 18 to 69 (70.4%) compared to patients aged 70+ (53.8%) and the difference was statistically significant (*P* = .045; Table 4). Likewise, patients who were concerned with knowing the level of training of their doctor were more likely to report the badges useful (70.7% vs 51.1%; *P* = .011).

Overall, 313 (74.0%) of the responders were satisfied with the amount of information received about the level of training of their care providers. The percentage of patients satisfied with this item was higher among patients with education of high school or less (88.8%), compared to the patients with higher education levels (73.5%, *P* < .001; Table 5).

**Table 5.** Patients’ Characteristics Associated With Satisfaction About the Amount of Information Received, and If the Badge Was Useful for Them.^a^

		Badge Helped Figure Out the Role of My Medical Providers					Satisfied With Information Received About Level of Care Providers’ Training				
		Agree		Disagree		*P* Value	Agree		Disagree		*P* Value
		N	(%)	N	(%)		N	(%)	N	(%)	
Age Group (70)	18-69	247	(70.4%)	104	(29.6%)	.045	274	(78.5%)	75	(21.5%)	.685
	70+	21	(53.8%)	18	(46.2%)		34	(82.9%)	7	(17.1%)	
Education level	High school or less	104	(72.2%)	40	(27.8%)	.491	127	(88.8%)	16	(11.2%)	<.001^b^
	Above high school	164	(68.3%)	76	(31.7%)		180	(73.5%)	65	(26.5%)	
Income levels	$0-$24 999	122	(92.2%)	47	(27.8%)	.790	142	(83.5%)	28	(16.5%)	.379
	$25,000 - $49 000	43	(76.8%)	13	(23.2%)		42	(77.8%)	12	(22.2%)	
	$50 000-$74 999	24	(68.6%)	11	(31.4%)		24	(66.7%)	12	(33.3%)	
	$75 000-$99 000	11	(73.3%)	4	(26.7%)		11	(73.3%)	4	(26.7%)	
	$100 000 +	28	(75.7%)	9	(24.3%)		31	(83.8%)	6	(16.2%)	
It is very important to me to know the level of training of my doctor	Agree	248	(70.7%)	103	(29.3%)	.011^c^	277	(78.7%)	75	(21.3%)	.704
	Disagree	24	(51.1%)	23	(48.9%)		35	(76.1%)	11	(23.9%)	
I have frequently watched medical shows	Agree	162	(71.1%)	66	(28.9%)	.272	182	(79.1%)	48	(20.9%)	.712
	Disagree	109	(65.7%)	57	(34.3%)		130	(77.4%)	38	(22.6%)	

^a^ n may be less than total N of 423 as some participants declined to answer some questions.

Chi-square test used to compare variables.

^b^ Chi-square = 12.87.

^c^ Chi-square = 6.48.

### Patients’ Perception of Medical Care Provider Roles

Overall, 71.6% (N = 303) of participants agreed that they knew the roles of their care providers in the ED. However, 50.5% (N = 153) of patients who agreed they knew the role of care providers did not know that “an attending doctor doesn’t require supervision by a resident”. Similarly, 19.1% (N = 58) of this group did not know that “a medical student could not write a prescription to send them home” (Table 6).

**Table 6.** Prevalence and Distribution of Patients’ Knowledge of Medical Care Providers’ Roles and Patients’ Levels of Satisfaction.

	I Knew the Roles of My Care Providers					I Was Satisfied With the Information My Providers Gave to Me Regarding Their Level of Training			
	Answer	Agree		Disagree		Agree		Disagree	
		N	(%)	N	(%)	N	(%)	N	(%)
A medical student is a medical doctor (MD)	Wrong/Don’t know	97	(32.0)	35	(32.4)	108	(34.5)	18	(20.9)
	Correct	206	(68.0)	73	(67.6)	205	(65.5)	68	(79.1)
An attending doctor requires supervision by a resident	Wrong/Don’t know	153	(50.5)	68	(63.0)	176	(56.2)	39	(45.3)
	Correct	150	(49.5)	40	(37.0)	137	(43.8)	47	(54.7)
Resident has completed medical school	Wrong/Don’t know	89	(29.4)	47	(43.5)	99	(31.6)	30	(34.9)
	Correct	214	(70.6)	61	(56.5)	214	(68.4)	56	(65.1)
Resident is the most highly trained doctor in ED	Wrong/Don’t know	146	(48.2)	54	(50.0)	163	(52.1)	31	(36.0)
	Correct	157	(51.8)	54	(50.0)	150	(47.9)	55	(64.0)
A resident requires no supervision when caring for patients	Wrong/Don’t know	158	(52.1)	59	(54.6)	173	(55.3)	38	(44.2)
	Correct	145	(47.9)	49	(45.4)	140	(44.7)	48	(55.8)
A medical student can write a prescription for you to go home with today	Wrong/Don’t know	58	(19.1)	31	(28.7)	65	(20.8)	21	(24.4)
	Correct	245	(80.9)	77	(71.3)	248	(79.2)	65	(75.6)
An attending has completed all medical training and requires no supervision	Wrong/Don’t know	93	(30.7)	37	(34.3)	95	(30.4)	30	(34.9)
	Correct	210	(69.3)	71	(65.7)	218	(69.6)	56	(65.1)
An attending doctor is the boss in the emergency department	Wrong/Don’t know	178	(58.7)	60	(55.6)	178	(56.9)	51	(59.3)
	Correct	125	(41.3)	48	(44.4)	135	(43.1)	35	(40.7)

Abbreviation: ED, emergency room.

## Discussion

The University of California, Irvine Medical Center, has gone to great lengths to clearly identify members of the patient care team with newly implemented badge system with the terms “medical student,” “resident,” and “attending.” However, it was unclear whether these titles are inherently obvious and understood by the patient population at our center. Our study found that a majority of patients felt they knew the roles of their medical care provider, but that on average patients answered few of the knowledge-based questions correctly, indicating a generally poor understanding of the medical training hierarchy. This is better than a previous study which showed only 44.5% knew their providers role [6]. However, our results show that many patients still did not know what that role meant. Again, this lack of understanding is consistent but slightly better than reported in previous studies [2].

Education was inversely associated with “satisfaction with amount of information received from care providers.” This may indicate lower demand for information among less educated patients. The application of this finding would be the need for specific straightforward creating plans for less educated patients, this could include proven methods of patient engagement such as motivational interviewing or teach-back techniques.

Badge buddies were considered to be useful and noticed by a majority of patients; however, based on the lack of understanding of the medical training hierarchy, it appears they are not enough to fully clarify the roles for patients. Patients may need more information about what each role means instead of just titles. Further research would be needed to evaluate if other methods such as videos explaining the different roles would be more useful for patients.

Furthermore, badge buddies may not be noticed or useful to everyone as we found that older patients did not notice them as often. However, those with increased concern regarding the level of training of their care providers noticed the badges more frequently suggesting these patients may have a better understanding or awareness of the different levels of training. Again, it is possible that another modality such as a videos, motivational interviews, or the teach-back method could be more effective and more accessible to patients of all ages [7–10].

Patients who watched medical drama and reality television shows did not have a statistically significant improvement of understanding of the medical training hierarchy compared to others. However, they did notice the badges more frequently suggesting they may be more aware of the terminology, even if that awareness does not translate to an understanding of what that terminology means.

A major limitation is that we are drawing conclusions about patient understanding of the medical training hierarchy based on 8 knowledge-based questions. We felt these questions would be representative of a patient’s general knowledge about various medical titles, although it is possible that we did not accurately capture this. However, our data are consistent with previous studies, wherein patients had a generally poor understanding of various medical titles. The ED is a stressful environment and it is possible that although all study participants were in stable condition and pending discharge, they did not correctly answer questions due to the nature of their circumstances.

## Conclusion

Our study found that the patient population at our teaching hospital has a generally poor understanding of the medical training hierarchy. Despite a growing trend for medical dramas and reality television shows depicting the ED and hospitals, viewership of these programs had no effect on patient understanding of various titles. Patients overwhelmingly felt that it was important for them to know the level of training of the medical staff interacting with them and felt that a large badge buddy clearly identifying provider roles was helpful. However, older patients benefited less from this intervention, and as such further research is needed to evaluate whether other interventions such as motivational interviewing, teach-back methods, or videos may improve understanding for a larger population of patients.

## Supplemental Material

Supplement_JPX - Patient Perception of Providers: Do Patients Understand Who Their Doctor Is? Click here for additional data file. Supplement_JPX for Patient Perception of Providers: Do Patients Understand Who Their Doctor Is? by Alisa Wray, Maja Feldman, Shannon Toohey, Andrew Wong, Wynne Breed, Emily Frisch, Soheil Saadat and Warren Wiechmann in Journal of Patient Experience
