# Supplementary material for: Patient Perception of Providers: Do Patients Understand Who Their Doctor Is?
Source: J Patient Exp. 2019 Dec 4;7(5):788–95. doi: 10.1177/2374373519892780 (PMC7705819; doi:10.1177/2374373519892780)
Supplement: Supplement_JPX - Patient Perception of Providers: Do Patients Understand Who Their Doctor Is? [file Supplement_JPX.pdf]

Supplement 1

Knowledge Based Questions

1. A medical student is a medical doctor (MD).
2. An attending doctor requires supervision by a resident.
3. A resident has completed medical school.
4. A resident is the most highly trained doctor in the emergency department.
5. A resident requires no supervision when caring for patients.
6. A medical student can write a prescription for you to go home with today.
7. An attending doctor has completed all medical training and requires no supervision when caring for patients.
8. An attending doctor is the boss in the emergency department.

Opinion Based Questions

1. It is very important to me to know the level of training of my doctor when I am being treated in the ED
2. I knew the roles of my care providers
3. The medical provider who cared for me identified him/herself
4. I could clearly see this badge on my medical providers
5. My medical providers wore this badge
6. I was satisfied with the amount of information my providers gave to me regarding their level of training
7. This badge helped me figure out the role of my medical providers
8. In the past, I have frequently watched medical shows on TV
9. Overall, I was satisfied with my visit today and the care I received by my medical team
